# Combined Transcriptome and Proteome Analysis of RpoS Regulon Reveals Its Role in Spoilage Potential of *Pseudomonas fluorescens*

**DOI:** 10.3389/fmicb.2019.00094

**Published:** 2019-02-06

**Authors:** Xiaoxiang Liu, Jun Xu, Junli Zhu, Peng Du, Aihua Sun

**Affiliations:** ^1^School of Basic Medical Sciences and Forensic Medicine, Hangzhou Medical College, Hangzhou, China; ^2^Hangzhou Lin'an District People's Hospital, Hangzhou, China; ^3^College of Food Science and Biotechnology, Zhejiang Gongshang University, Hangzhou, China

**Keywords:** transcriptome, proteome, RpoS, regulon, *Pseudomonas fluorescens*, food spoilage

## Abstract

Microbial contamination is considered the main cause of food spoilage. *Pseudomonas fluorescens* is a typical spoilage bacterium contributing to a large extent to the spoilage process of proteinaceous foods. RpoS is known as an alternative sigma factor controlling stress resistance and virulence in many pathogens. Our previous work revealed that RpoS contributes to the spoilage activities of *P*. *fluorescens* by regulating resistance to different stress conditions, extracellular acylated homoserine lactone (AHL) levels, extracellular protease and total volatile basic nitrogen (TVB-N) production. However, RpoS-dependent genes in *P*. *fluorescens* remained undefined. RNA-seq transcriptomics analysis combined with quantitative proteomics analysis based on multiplexed isobaric tandem mass tag (TMT) labeling was performed in the *P*. *fluorescens* wild-type strain UK4 and its derivative carrying an *rpoS* mutation. A total of 375 differentially expressed coding sequences (DECs) and 212 differentially expressed proteins (DEPs) were identified. The DECs were further verified by qRT-PCR. The combined transcriptome and proteome analyses revealed the involvement of this regulator in several cellular processes, mainly including polysaccharide metabolism, intracellular secretion, extracellular structures, cell wall biogenesis, stress responses, and amino acid and biogenic amine metabolism, which may contribute to the biofilm formation, stress resistance, and spoilage activities of *P*. *fluorescens*. Moreover, we indeed observed that RpoS contributed to the production of the macrocolony biofilm's matrix. Our results provide insights into the regulatory network of RpoS and expand the knowledge about the role of RpoS in the functioning of *P. fluorescens* in food spoilage.

## Introduction

Most food products are highly perishable as they contain rich nutrient contents for microbial development. *Pseudomonas fluorescens* is a common spoilage microorganism in proteinaceous raw foods stored under aerobic refrigerated conditions, such as dairy products (Andreani et al., 2015), meat (Remenant et al., 2015), and seafoods (Xie et al., 2018). As a spoiler, *P*. *fluorescens* can produce ammonia, amine, ketones, aldehydes, esters, organic acids and non-H_2_S sulfides with spoilage off-odors and off-flavors (Ghaly et al., 2010). Moreover, it also causes spoilage by producing heat-stable lipases and proteases (Rajmohan et al., 2002), biofilms (Aswathanarayan and Vittal, 2014), biosurfactants (Mellor et al., 2011), siderophores (Liu et al., 2017), pigments (Andreani et al., 2014), and quorum-sensing signaling molecules (Liu et al., 2018). So far, the knowledge of regulatory mechanisms of bacterial spoilage is still very limited.

RpoS is an alternative sigma factor of RNA polymerase that was first described in *Escherichia coli* (Hengge-Aronis, 1999). Homologs of RpoS have also been characterized in the γ, β, and δ subclasses of *Proteobacteria* (Núñez et al., 2006; Dong and Schellhorn, 2010). A sigma factor is needed by the RNA core polymerase to recognize promoters and initiate transcription. In addition to housekeeping sigma factors controlling the transcription of the majority of genes, including essential genes, bacteria possess alternative sigma factors that recognize specific set of promoters (Schellhorn, 2014). RpoS is an alternative sigma factor induced in stationary growth phase and under stress conditions (Landini et al., 2014). Consequently, *rpoS* deficient mutants are sensitive to nutrient starvation, heat, osmolarity, acidic pH, oxidative stress, and are usually altered for virulence (Dong and Schellhorn, 2010). RpoS regulates the production of alginate, exotoxin A, and secreted proteases in *Pseudomonas aeruginosa* (Suh et al., 1999; Sonnleitner et al., 2003); the formation of virulence factor curli in *E. coli* (Mika and Hengge, 2014); the secretion of extracellular proteases and siderophores in *Burkholderia pseudomallei* (Wongtrakoongate et al., 2012); and the generation of flagella, exopolysaccharides, and biofilms in *Yersinia pseudotuberculosis* (Guan et al., 2015). The involvement of RpoS in stress resistance and virulence suggests that RpoS may play a key role in modulating the spoilage activity of *P*. *fluorescens* in food systems. Our previous work revealed that RpoS contributes to spoilage potential in *P*. *fluorescens* by regulating resistance to different stress conditions, extracellular AHL levels, extracellular proteases and TVB-N production (Liu et al., 2018). However, it remains unknown how many genes are regulated by this transcriptional regulator.

RpoS regulons have been characterized by microarrays in *E*. *coli* and *P*. *aeruginosa*. RpoS controls a large regulon consisting of 10% of the genome in the *E*. *coli* strain K12 in the stationary phase and under stress conditions (Patten et al., 2004; Dong and Schellhorn, 2009). In *P*. *aeruginosa*, 772 genes are regulated by RpoS in the stationary but not in the logarithmic growth phase, including over 40% of all genes controlled by quorum sensing (Schuster et al., 2004). In addition, the RpoS regulon of *B. pseudomallei* has been identified using a proteomics approach, and 70 differentially expressed proteins were identified (Osiriphun et al., 2009). RNA sequencing (RNA-seq) is an attractive method to monitor global transcriptomic changes, overcoming many defects of traditional DNA microarrays (Marioni et al., 2008). Proteomic sequencing is an important technique to explore changes in gene expression at protein levels. The latest protein quantitative analysis technology based on mass spectrometry (MS) and multiplexed isobaric TMT labeling is widely used (Ahrné et al., 2016; Han, 2017). Thus, combining the two techniques should substantially improve the global view of the regulatory roles of RpoS.

To better understand the role of RpoS in the spoilage activity of *P*. *fluorescens*, we combined transcriptome and proteome analyses in the *P*. *fluorescens* wild-type strain UK4 and the *rpoS* mutant in stationary phase to identify the RpoS regulon. We found that RpoS influenced the expression of a large number of CDSs at the mRNA and protein levels. The DECs were verified by quantitative real-time PCR (qRT-PCR). The function of the RpoS regulon was further analyzed by COG (cluster of orthologous groups) categorization, GO (gene ontology) enrichment, and KEGG (Kyoto encyclopedia of genes and genomes) enrichment. Our study revealed the large impact of RpoS on gene expression at the mRNA level and protein level, providing insights into the spoilage function of *rpoS* in *P*. *fluorescens*.

## Materials and Methods

### Bacterial Strains and Culture Conditions

The wild-type strain UK4 and the *rpoS* in-frame deletion mutant of *P*. *fluorescens* (Liu et al., 2018) were grown aerobically at 28°C with shaking at 220 rpm in nutrient broth (NB) medium, and growth was monitored spectrophotometrically at OD_600_. Cultures were grown in triplicates and the bacterial cells at stationary-phase (OD_600_ ≈ 1.5) were harvested by centrifugation at 7,000 g for 10 min, then frozen in liquid nitrogen immediately for RNA and protein isolation.

### Illumina Library Preparation and RNA-seq

Three biological replicates were prepared for the wild-type strain and the *rpoS* mutant. The total RNA was isolated using an RNeasy Plant Mini Kit (Qiagen, Germany) and was treated with an RNase-Free DNase Set (Qiagen, Germany) to remove the contaminating DNA. The concentration and quality were determined using a NanoDrop spectrophotometer (Thermo, USA) and Agilent 2100 Bioanalyzer (Agilent, USA). Then, rRNA from the total RNAs was removed using a Ribo-Zero Magnetic Kit (Epicentre, USA) for Gram-negative bacteria as per the manufacturer's protocol. Fragmentation buffer was added to cleave the mRNA into short fragments. Strand-specific RNA sequencing libraries were constructed according to a modified deoxy-UTP (dUTP) strand-marking protocol as described previously (Borodina et al., 2011). Briefly, first strand cDNA was synthesized using random hexamer primers and M-MuLV Reverse Transcriptase (NEB, USA). Double-stranded cDNA was subsequently synthesized with dUTP incorporation into the second strand. Illumina TruSeq adaptors were ligated to the ends of the cDNA fragments, and then amplified with the adaptor primers according to the manufacturer's instructions (Illumina, USA). The fragments were purified with an AMPure XP system (Beckman Coulter, USA) to select cDNA fragments of preferentially 150~200 bp in length. Then the dUTP-marked strand was selectively degraded using USER enzyme (NEB, USA) and the remaining strand was amplified to produce a cDNA library suitable for sequencing. Following validation with the Agilent Bioanalyzer 2100 system (Agilent, USA), the cDNA library was sequenced on a flow cell using high-throughput 150-bp pair-end mode on an Illumina HiSeq 4000 platform (Illumina, USA).

### Analysis of RNA-seq Data

The raw sequencing data of RNA-seq was deposited to the Short Reads Archive of NCBI with accession numbers SRP158301 (https://www.ncbi.nlm.nih.gov/sra/). Clean data (clean reads) were obtained by removing adapter-containing reads, higher N rate reads (N rates > 10%), and low-quality reads (50% bases with Q-score ≤ 5) from the raw data (raw reads) using in-house Perl scripts. At the same time, the Q20, Q30 and GC content of the clean reads were calculated. Of the clean reads, the Q20 and Q30 values were > 98 and 95%, respectively, showing that the data were of high quality (Supplementary Table S1). All downstream analyses were based on the high-quality clean data. Sequence alignment with the reference UK4 genome sequence (Dueholm et al., 2014) was conducted by Bowtie2-2.2.3 (Langmead and Salzberg, 2012). The software parameter mismatch was set to 2, and other parameters were default values. Subsequent analyses were based on the unique mapped reads. For gene expression analysis, HTSeq v0.6.1 was used to count the read numbers mapped to each gene. And then FPKM (Fragments Per Kilobase of transcript sequence per Millions base pairs sequenced) of each gene was calculated (Trapnell et al., 2012). The FPKM value was directly used for comparing differences in gene expression among samples, as this method removes effects caused by sequencing depth and gene length in calculating gene expression. The CDSs with FPKM > 1 were considered to be expressed. The software package DESeq R 1.18.0 was used to calculate the fold-change of transcripts and to screen all DECs (Wang et al., 2010). DESeq provides statistical routines for determining differential expression in digital CDS expression data using a model based on the negative binomial distribution. The resulting *p*-values were adjusted using Benjamini and Hochberg's approach for controlling the false discovery rate. The criteria of significant difference expression were |log_2_ fold change| ≥1 and adjusted *p*-value (padj) ≤ 0.05. Putative operons analysis are based on chromosomal organization and similarity of transcript patterns. The criteria used to generate a list of potential operons were that every CDS within a gene cluster was in the same orientation, that every CDS had the same trend in differential expression, that the absolute transcript profiles of the candidate CDSs in an operon showed patterns similar to each other, and that the intergenic region between two adjacent CDSs was <250 bp (Schuster et al., 2004).

### Protein Extraction and LC-MS/MS Analysis

Protein extraction and LC-MS/MS analysis were performed using the protocol described previously (Guerreiro et al., 2014; Peng et al., 2018) with a few modifications. Briefly, a sample was sonicated three times on ice in lysis buffer containing 8 M urea, 2 mM EDTA, 10 mM DTT, and 1% Protease Inhibitor Cocktail (Sigma-Aldrich, USA). After centrifugation at 20,000 g at 4°C for 10 min, the debris was removed, and the supernatant was precipitated with cold 15% trichloroacetic acid for 4 h at −20°C. After centrifugation at 4°C for 3 min, the obtained precipitate was washed with cold acetone three times. The protein precipitate was redissolved in buffer (8 M urea, 100 mM TEAB, pH 8.0), and the protein concentration in the supernatant was determined by a 2-D Quant kit (GE Healthcare, USA) according to the manufacturer's protocol. The protein solution was reduced with 5 mM DTT for 30 min at 56°C and alkylated with 11 mM iodoacetamide for 15 min at room temperature in the dark. For trypsin digestion, the protein sample was diluted with buffer (200 mM TEAB, < 2 M urea). Finally, trypsin was added to approximately 100 μg protein for each sample at an enzyme:protein ratio of 1:50 for the first overnight digestion and an enzyme:protein ratio of 1:100 for a second 4-h digestion. After trypsin digestion, peptides were desalted by a Strata X C18 SPE column (Phenomenex, USA) and vacuum-dried. The digested peptides were reconstituted in 1 M TEAB and were labeled using a 6-plex TMT kit (Thermo, USA) according to the manufacturer's instructions to perform protein quantitation. The peptides from the six samples of UK4 and the *rpoS* mutant were labeled with 126, 127, 128, 129, 130, and 131 TMT reagents, respectively. Each sample was mixed and then fractionated using high pH reverse-phase HPLC with an Agilent 300 Extend C18 column (5 μm particles, 4.6 mm inner diameter, 250 mm length). Briefly, peptides were first separated with a gradient of 2–60% acetonitrile in 10 mM ammonium bicarbonate (pH 9.0) over 80 min into 80 fractions. Then, the peptides were combined into 18 fractions and dried by vacuum centrifugation. For LC-MS/MS analysis, peptides were dissolved in 0.1% formic acid, and loaded onto a reversed-phase pre-column directly (Acclaim PepMap 100, Thermo, USA). Peptide separation was carried out using a reversed-phase analytical column (Acclaim PepMap RSLC, Thermo, USA). The gradient was comprised of an increase from 5 to 25% of solvent B (0.1% formic acid in 98% acetonitrile) over 26 min, 25–40% over 8 min, 80% over 3 min, then held at 80% for the last 3 min, all at a constant flow rate of 350 nl/min on an EASY-nLC 1000 UPLC system (Thermo, USA). The peptides were subjected to nanoelectrospray ionization followed by tandem MS in Q Exactive (Thermo, USA) coupled online to the UPLC. Intact peptides were detected in the orbitrap at a resolution of 70,000. Peptides were selected for MS/MS using Normalized Collision Energy (NCE) setting as 28. Ion fragments were detected in the orbitrap at a resolution of 17,500. Three biological replicates for each strain were used for proteomic analyses.

### Proteomics Data Processing

The MS proteomics data was deposited to the ProteomeXchange Consortium with the dataset identifier PXD010845 (https://www.ebi.ac.uk/pride/archive/). The MS/MS data was processed using MaxQuant with the integrated Andromeda search engine (v.1.5.2.8). Tandem mass spectra were searched against the genome database of *P*. *fluorescens* UK4 containing 5321 proteins in GenBank (https://www.ncbi.nlm.nih.gov/genome/150?genome_assembly_id=204445). Trypsin was chosen as cleavage specificity allowing up to two missing cleavages. Carbamidomethyl on Cys was specified as a fixed modification, while oxidation on Met and acetylation on protein N-term were specified as variable modifications. Mass error was set to 10 ppm for precursor ions and 0.02 Da for fragment ions. For protein quantitation, protein quantitative ratios were weighted and normalized relative to the median ratio in Mascot (http://www.matrixscience.com). Only proteins with significant quantitative ratios between the two strains (*p* < 0.05) and with fold changes >1.2 or <0.83 were considered to be differentially expressed (Ning et al., 2018).

### qRT-PCR

To confirm the RNA-Seq results, 30 DECs from the RNA-Seq analysis were selected, and qRT-PCR was carried out to verify the expression changes of these 30 DECs. The qRT-PCRs were performed with RNA samples used for RNA-seq. The total RNA was reverse transcribed to single-stranded cDNA using a hexamer primer and SuperScript III First-Strand Synthesis SuperMix (Invitrogen, USA). Quantitative real-time PCR was performed on a CFX384 Touch Real-Time PCR Detection System (Bio-Rad, USA) using Power SYBR1 Green PCR Master Mix (Applied Biosystems, USA) according to the manufacturers' instructions. Gene-specific primers are listed in Supplementary Table S2, and the 16S rRNA gene was used as the internal control to normalize mRNA abundance between samples (Leneveu-Jenvrin et al., 2015). PCR was performed according to the following steps (two-step PCR amplification procedure): 1 min at 95°C, 40 cycles of 15 s at 95°C, and 25 s at 63°C. Melting curve analysis of amplification products was performed to evaluate the specificity of the amplification. The relative expression was calculated using the 2^−ΔΔ*Ct*^ method (Livak and Schmittgen, 2001). Samples were run in triplicate, and the experiments were repeated at least three times.

### COG Categorization

Function description of DECs and DEPs was performed according to a *P*. *fluorescens* UK4 genome annotation (http://www.pseudomonas.com/) and relative reports. COG functional categories for the DECs and DEPs were conducted according to the NCBI COG database (http://www.ncbi.nlm.nih.gov/COG/) and the function descriptions.

### GO and KEGG Enrichment Analysis

The GO and KEGG pathway annotations for DECs and DEPs were performed using the UniProt-GOA database (http://www.ebi.ac.uk/GOA/) and KEGG database (http://www.kegg.jp/kegg/pathway.html). A two-tailed Fisher's exact test was employed to test the enrichment of the DECs and DEPs against all identified genes and proteins. Corrections for multiple hypothesis testing were carried out using standard false discovery rate control methods. GO and KEGG pathways with *p* < 0.05 were considered significant.

### Transmission Electron Microscopy (TEM) of Macrocolony Biofilm

The wild-type strain UK4 and the *rpoS* mutant were grown overnight in liquid NB medium under aeration at 28°C. A total of 5 μl of the overnight cultures was spotted on Congo red plates (1% tryptone, 1% agar, 20 μg/ml Congo red, and 10 μg/ml Coomassie brilliant blue G250) (Friedman and Kolter, 2004). Plates incubated at 28°C for up to 7 days were used to judge colony morphology and color. For TEM, the macrocolonies were scraped from the agar plates (without Congo red and Coomassie brilliant blue G250) gently and were fixed with 2.5% glutaraldehyde for 2 h at room temperature, then the macrocolonies were postfixed, dehydrated, embedded in TAAB resin, ultrathin sectioned, and stained according to the protocol previously described (Zhang et al., 2012). The macrocolony biofilms were observed under a transmission electron microscope (Hitachi H-600, Japan).

## Results

### Overview of Transcriptomic and Proteomic Data

Our previous studies suggested that RpoS plays a global regulatory role in the stress resistance, quorum sensing and spoilage potential of *P*. *fluorescens*. In the present study, as a further step to investigate the regulatory role of RpoS, we applied combined transcriptome and proteome analyses to establish the set of CDSs or proteins belonging to the RpoS regulon. We compared *P*. *fluorescens* wild-type strain UK4 with its derivative, the *rpoS* mutant, which has a 705 bp segment deletion inside the *rpoS* coding region (Liu et al., 2018). For transcriptome analysis, three RNA-Seq libraries were prepared for each strain grown to the stationary phase under the same conditions. After filtering through the raw reads, there were on average 17,442,829 clean reads for UK4 and 18,902,146 for the mutant, giving rise to an average 2.62 G and 2.84 G of total clean bases, respectively (Supplementary Table S1). The clean reads of the *P*. *fluorescens* transcriptome were mapped to the genome sequence of UK4. The unique mapped rates to the genome were at least 97.7% in all samples (Supplementary Table S3). In the genome of UK4, 98.90% of CDSs were quantified. All the detailed data of the CDSs from RNA-seq are shown in Supplementary Table S4. For proteome analysis, a total of 135,890 spectra generated from 3 biological replicates of UK4 and the *rpoS* mutant were analyzed using the Andromeda search engine. There were 35,804 spectra matched with proteins of UK4, comprising 19,399 peptides and 19,345 unique peptides. Finally, 2730 protein were identified, among which 2,559 proteins were quantified. The detailed information of protein MS data are shown in Supplementary Table S5.

### Identification of DECs and DEPs

**Figure 1:**
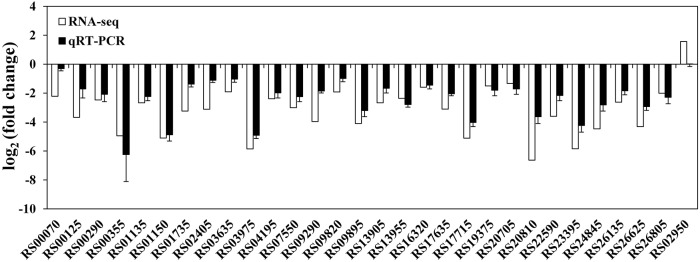
Validation of RNA-seq data using qRT-PCR. White bars represent RNA-seq data, while the black bars represent mean values of log_2_ (fold change) observed for the *rpoS* mutant samples vs. the wild-type samples. The qRT-PCR results are the mean of three biological replicates with three technical replicates for each gene. Error bars represent standard deviation.

At the mRNA level, a total of 372 DECs were identified using the statistical criteria (|log_2_ fold change| ≥1, padj ≤0.05). Among these DECs, 352 were significantly downregulated and 20 were significantly upregulated in the *rpoS* mutant compared to the wild-type strain (Supplementary Figure S1), and 155 DECs were found to be organized in 50 supposed operons. The CDS locus tags, putative operon organization, readcounts, log_2_ (fold change), padj, CDS product descriptions, COG protein categories, and GO and KEGG annotations of the DECs are provided in Supplementary Table S6. To validate the data generated from the RNA-seq experiment, 29 downregulated and 1 upregulated CDSs were randomly selected from the DECs to verify their expression by qRT-PCR. The qRT-PCR results agreed with RNA-seq data, except for CDS RS02950 (Figure 1), indicating that our RNA-seq results are reliable.

At protein levels, proteins with significant quantitative ratios between the *rpoS* mutant and the wild-type strain (*p* < 0.05) and with fold changes > 1.2 or <0.83 were considered as DEPs. In brief, a total of 212 DEPs were identified, of which, 140 proteins were downregulated in the *rpoS* mutant, and 72 proteins were upregulated (Supplementary Figure S2; Supplementary Table S7).

**Figure 2:**
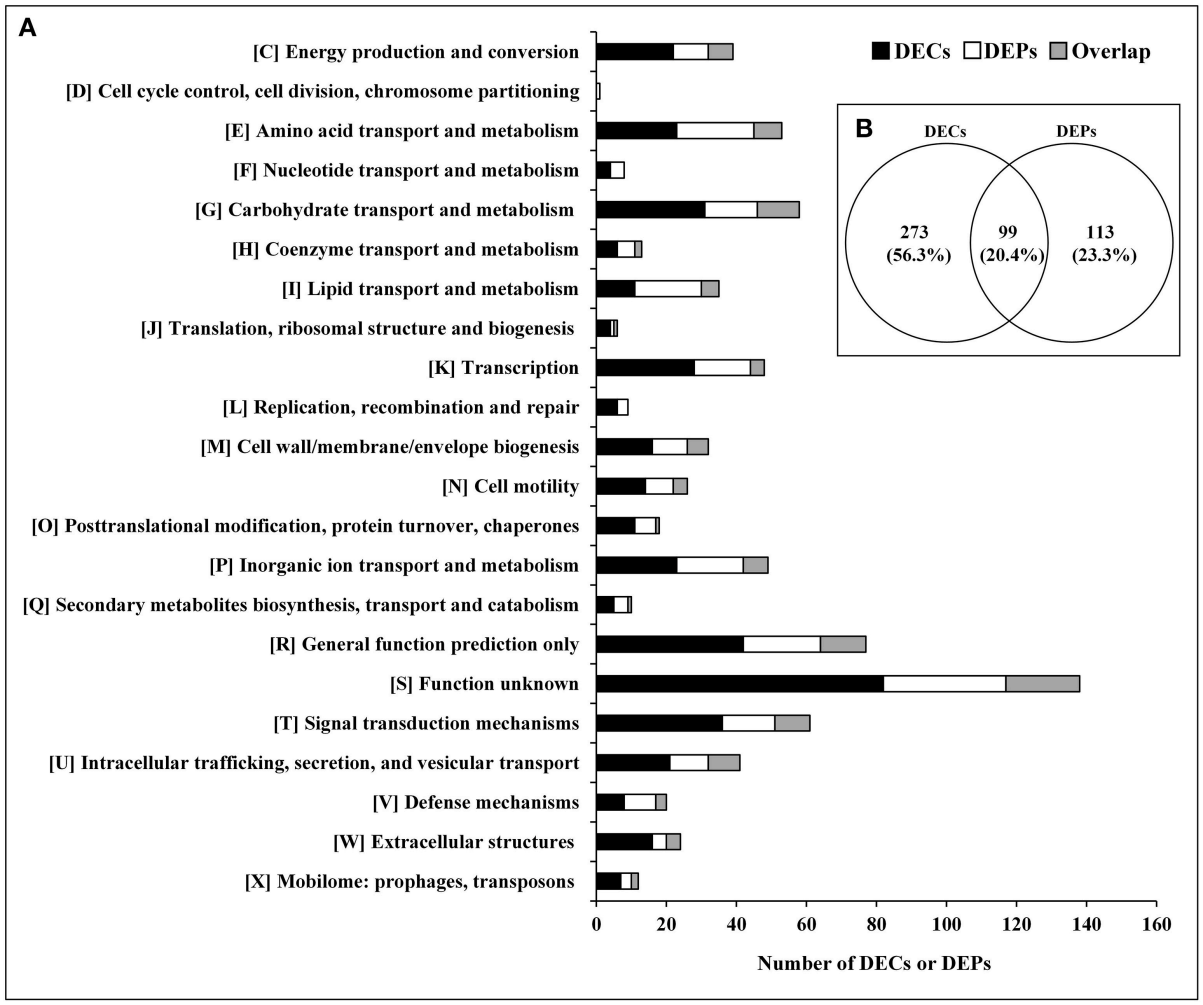
DECs and DEPs in the *rpoS* mutant in relation to the wild-type strain UK4. **(A)** Classification of the DECs, DEPs, and their overlap into individual functional groups (COGs C-X). **(B)** Venn diagram of DECs and DEPs. This figure was drawn using Venny 2.0 (http://bioinfogp.cnb.csic.es/tools/venny/index.html).

The relationship between the transcriptome and proteome data were further analyzed. The 372 DECs and 212 DEPs identified were used to perform correlation analysis, and a total of 99 candidates were obtained in the overlap of transcriptome and proteome datasets (Figure 2; Supplementary Table S8). The 99 DEPs (46.7% of the total DEPs) were consistent with the tendency of the change in abundance of the mRNA. In general, in both bacteria and eukaryotes, mRNA formation is only partially correlated with protein synthesis (Maier et al., 2011; Vogel and Marcotte, 2012; Lv et al., 2017). Maybe it is because the RNA-seq detection is much more sensitive than proteome determination, or because of post-transcriptional, translational and protein degradation regulation (Maier et al., 2009; Zhang et al., 2018). Thus, we can obtain useful insight that is otherwise impossible to obtain from individual analysis of mRNA or protein expressions by combing analysis of transcriptomic and proteomic data (Oshone et al., 2017).

### Functional Analysis of DECs and DEPs

The DECs and DEPs were classified using their functional annotations in GenBank or through homology with protein functions determined from the COG database (Figure 2). COG functional analysis assigned DECs and DEPs to 22 predicted pathways. According to the number of the DECs and DEPs, except for groups with function unknown (COG S) and general function prediction only (COG R), the top 5 groups were related to signal transduction mechanisms (COG T), carbohydrate transport and metabolism (COG G), amino acid transport and metabolism (COG E), transcription (COG K), and inorganic ion transport and metabolism (COG P).

**Figure 3:**
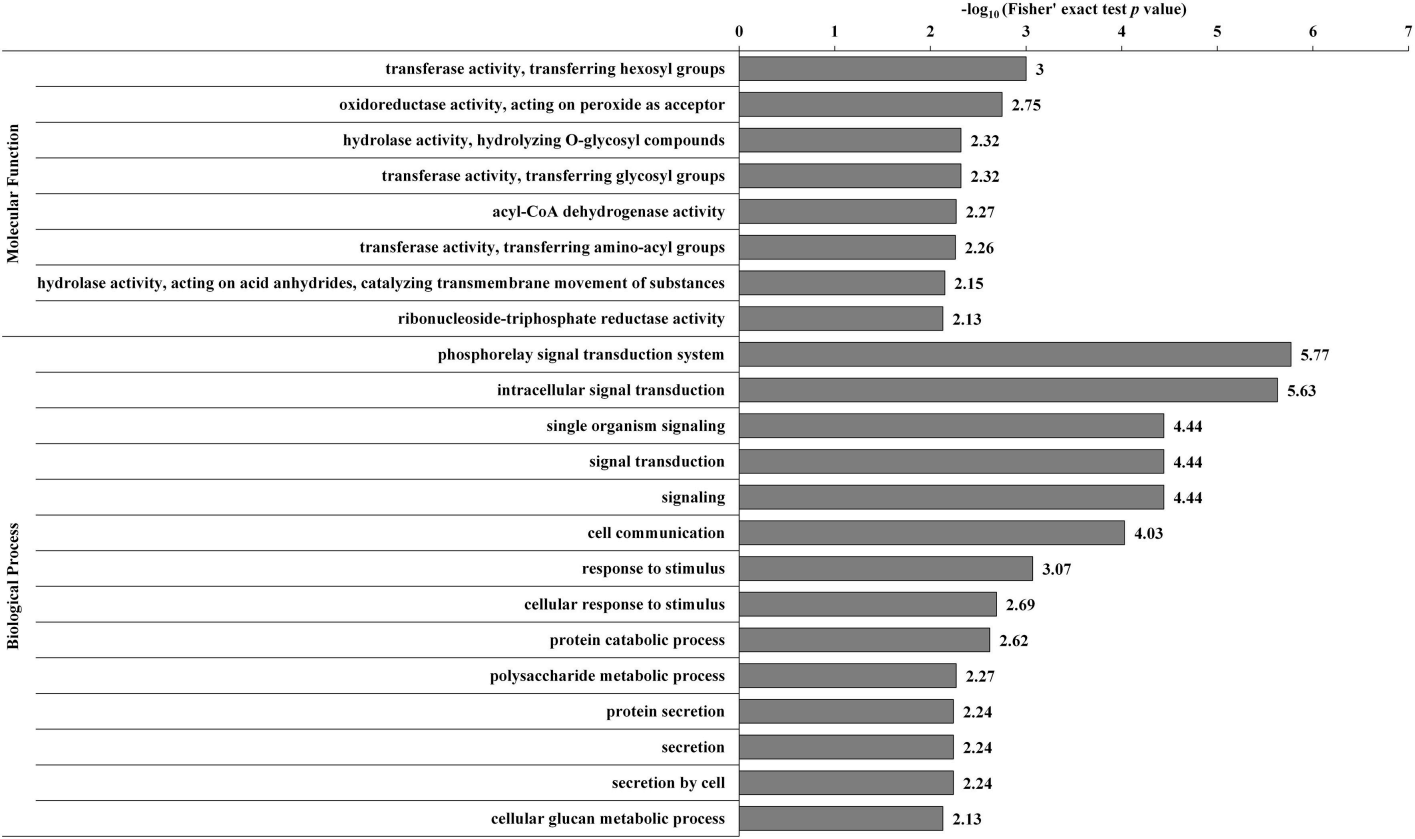
GO-based enrichment analysis of the combined DECs and DEPs. GO categories significantly enriched (*p* < 0.05) are shown in the figure.

The DECs and DEPs were combined, and GO enrichment analysis was performed (Supplementary Table S9). The significantly enriched GO functions are shown in Figure 3. At the biological process level, the DECs and DEPs were mainly involved in the phosphorelay signal transduction system, cell communication, response to stimulus, polysaccharide metabolic process, and protein secretion. At the molecular function level, the DECs and DEPs were mainly enriched in transferase activity transferring hexosyl groups, oxidoreductase activity acting on peroxide as an acceptor, and hydrolase activity hydrolyzing O-glycosyl compounds.

**Figure 4:**
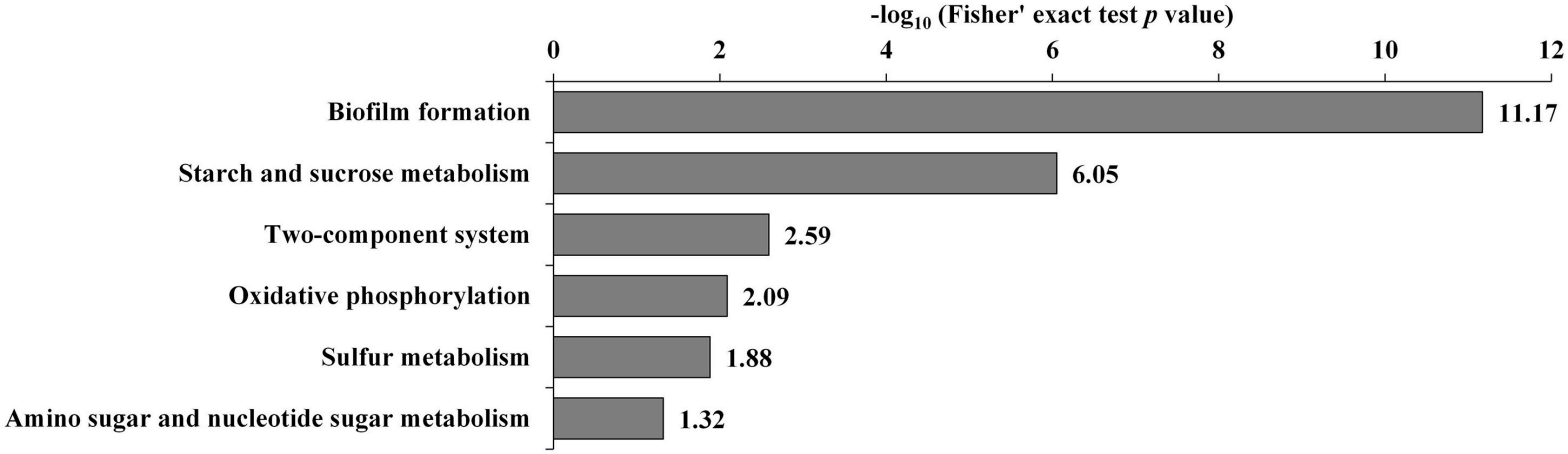
KEGG pathway enrichment analysis of the combined DECs and DEPs. KEGG pathways significantly enriched (*p* < 0.05) are shown in the figure.

**Figure 5:**
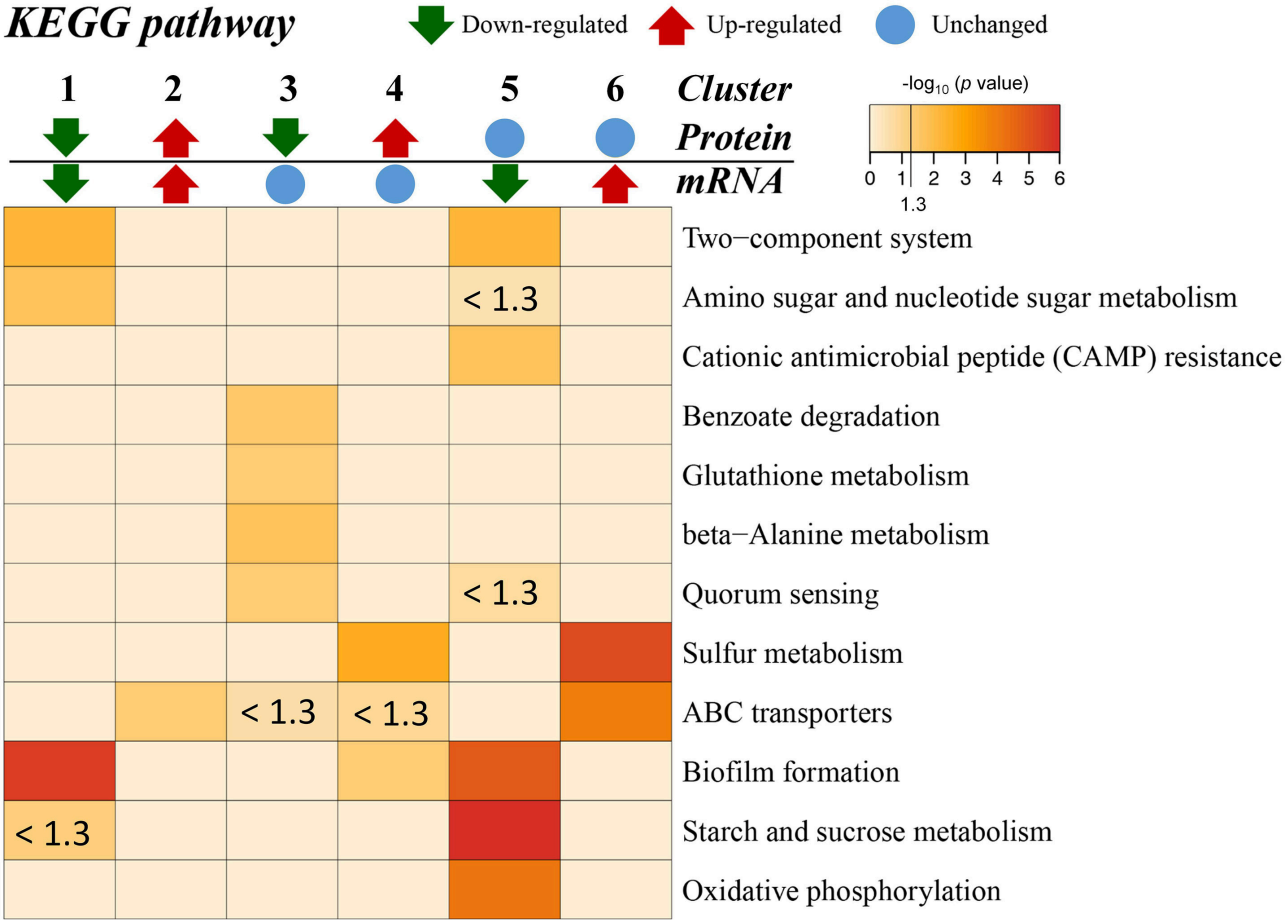
KEGG pathway enrichment for different categories based on protein-mRNA regulation types. 1, the cluster with protein and mRNA both downregulated in the mutant; 2, the cluster with protein and mRNA both upregulated in the mutant; 3, the cluster with only protein downregulated in the mutant; 4, the cluster with only protein upregulated in the mutant; 5, the cluster with only mRNA downregulated in the mutant; 6, the cluster with only mRNA upregulated in the mutant. The pathways with *p* < 0.05 [–log_10_ (*p*) > 1.3] are considered significant. The color intensity presents the enrichment level as shown in the bar of –log_10_ (*p*). The deep red color codes represent the remarkable enriched pathways. The pale yellow in background represents there is no enrichment in the corresponding pathway.

Additionally, the transcriptome and proteome datasets were also used for KEGG pathway enrichment analysis. All the DECs and DEPs were combined and were significantly enriched in six pathways, including biofilm formation, starch and sucrose metabolism, two-component system, oxidative phosphorylation, sulfur metabolism, and amino sugar and nucleotide sugar metabolism (Figure 4; Supplementary Table S10). In addition, the combined DECs and DEPs were divided into six categories according to their protein-mRNA regulation types (Figure 5; Supplementary Table S10). In the cluster with protein and mRNA both downregulated in the mutant, the significantly enriched pathways were biofilm formation, two-component system and amino sugar and nucleotide sugar metabolism. In the cluster with protein and mRNA both upregulated, only the pathway ABC transporters were significantly enriched. In the cluster with only protein downregulated, the functions were mainly enriched in benzoate degradation, glutathione metabolism, beta-alanine metabolism, and quorum sensing. In the cluster with only protein upregulated, the two pathways, sulfur metabolism and biofilm formation, were significantly enriched. In the cluster with only mRNA downregulated, the functions were mainly enriched in starch and sucrose metabolism, biofilm formation, oxidative phosphorylation, two-component system and cationic antimicrobial peptide resistance. In the cluster with only mRNA upregulated, the functions were mainly involved in sulfur metabolism and ABC transporters.

### Effects of *rpoS* Mutation on Macrocolony Biofilm Formation

**Figure 6:**
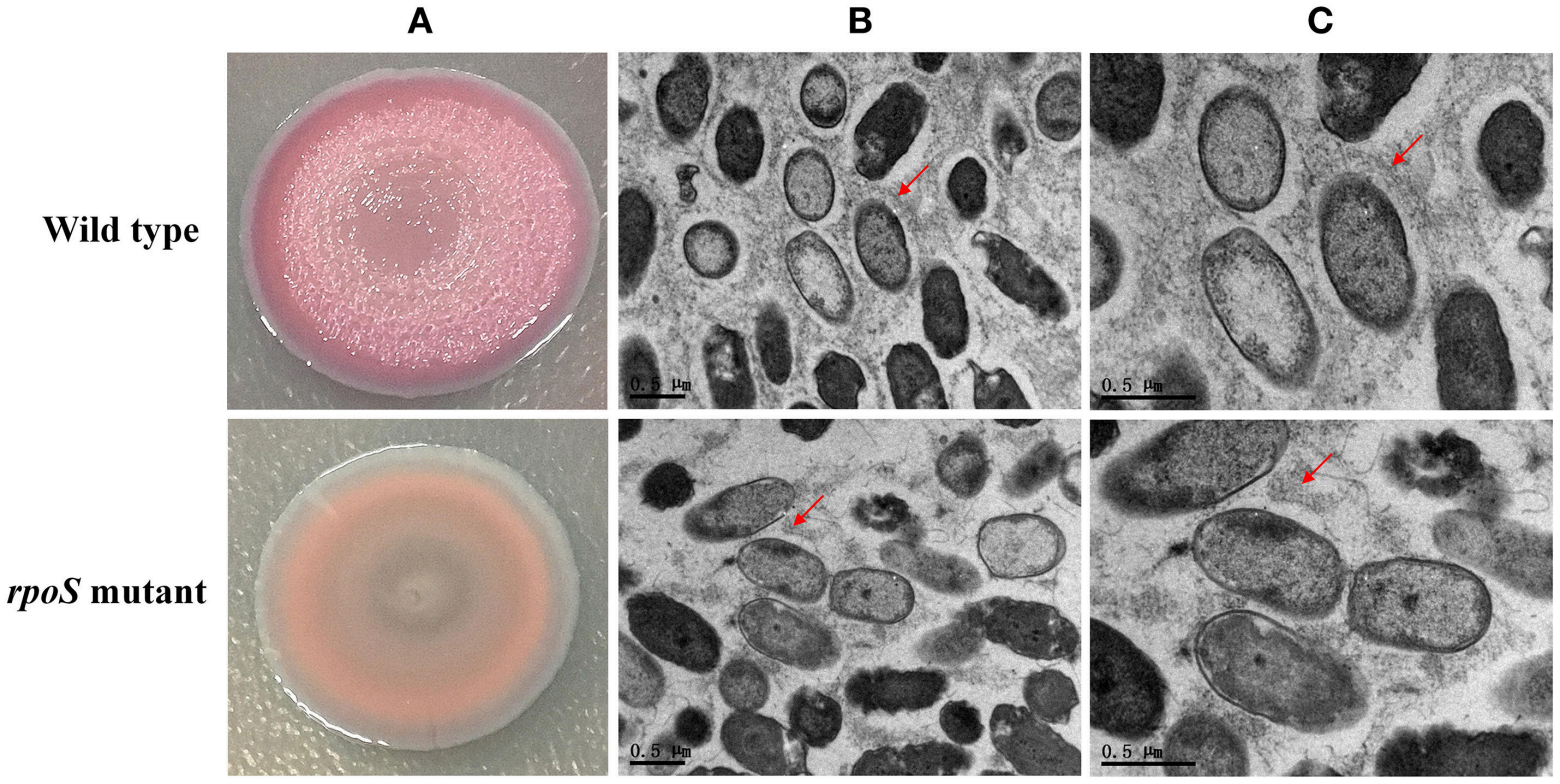
Macrocolony biofilm properties of the *rpoS* mutant in comparison to the wild-type. **(A)** Macrocolony morphology of the wild-type and the *rpoS* mutant grown on Congo red and Coomassie brilliant blue plates for 7 days. **(B,C)** Transmission electron micrographs of the macrocolony biofilms at ×11,500 and ×20,500 magnification. Matrix materials surrounding the cells are marked with red arrows.

Macrocolony biofilms that form on nutrient-providing semi-solid agar plates reflect the conditions of biofilms that grow on decaying organic materials such as soil or human food. We compared macrocolony biofilms of the *rpoS* mutant and the wild-type. Macrocolonies of the strains were grown for 3–7 d at 28°C on agar plates containing Congo red. Seven-day-old macrocolony are shown in Figure 6. The wild-type strains formed red and wrinkled macrocolonies whereas the *rpoS* mutant macrocolonies were pale pink and smooth. In addition, we used TEM to visualize the macrocolonies at the cellular level. Although both colonies of the wild-type and the mutant contained rod-shaped cells, it was apparent that the wild-type cells were embedded in an extracellular matrix that formed basket-like networks whereas the extracellular matrix of the mutant cells was disordered and of lower content than the wild-type. These results suggest that RpoS is involved in the production of the biofilm matrix.

## Discussion

Our previous work showed that the spoilage activities of *P*. *fluorescens* were regulated by RpoS. To better understand its network, mechanisms, and association with spoilage activities, we report here for the first time, the initial characterization of the RpoS regulon in an important spoilage bacterium *P*. *fluorescens*, via combined transcriptomic and proteomic analyses. In this section, we discuss the most interesting pathways regulated by RpoS. The representative DECs and DEPs are listed in Table 1.

**Table 1:** Representative CDSs differentially expressed in the *rpoS* mutant and the wild-type strain UK4 at the mRNA and protein levels.

**Locus tag^a^**	**Protein accession**	**Function description**	**mRNA Δ*rpoS*/UK4 ratio (adjusted *p*-value)^b^**	**Protein Δ*rpoS*/UK4 ratio (*p*-value)^c^**	**Regulation type (mRNA/protein)^d^**
**TRANSCRIPTION FACTORS AND SIGNAL TRANSDUCTION**					
HZ99_RS01135	WP_038440714.1	Acyl-homoserine-lactone synthase RhlI	0.157 (2.51E-12)	–	Down/–
HZ99_RS01140	WP_038440716.1	Transcriptional regulator RhlR	0.192 (2.38E-10)	–	Down/–
HZ99_RS01145	WP_038440719.1	Rhamnosyltransferase chain B, RhlB	0.034 (1.36E-24)	–	Down/–
HZ99_RS01150	WP_038440722.1	Rhamnosyltransferase 1 subunit A, RhlA	0.029 (3.31E-19)	–	Down/–
HZ99_RS01735	WP_038440888.1	LuxR family transcriptional regulator	0.106 (1.78E-04)	–	Down/–
HZ99_RS01825	WP_038440917.1	RNA polymerase sigma factor	0.176 (4.49E-05)	–	Down/–
HZ99_RS03980	WP_038441499.1	Fis family transcriptional regulator	0.083 (4.47E-05)	–	Down/–
HZ99_RS09360	WP_038442561.1	DNA-binding transcriptional regulator BasR (PmrA)	0.232 (7.59E-08)	–	Down/–
HZ99_RS09580	WP_003178872.1	Carbon storage regulator RsmA	0.262 (6.78E-07)	–	Down/–
HZ99_RS09820	WP_038442724.1	RNA polymerase subunit sigma-24, RpoE	0.265 (4.04E-02)	–	Down/–
HZ99_RS09895	WP_038442747.1	Methyl-accepting chemotaxis protein	0.058 (1.43E-03)	0.291 (4.20E-05)	Down/Down
HZ99_RS10650	WP_038442895.1	Diguanylate cyclase response regulator WspR	0.329 (6.95E-03)	–	Down/–
HZ99_RS10655	WP_038442896.1	Chemotaxis-specific methylesterase WspF	0.280 (1.11E-06)	0.746 (3.24E-02)	Down / Down
HZ99_RS10660	WP_038442897.1	Chemotaxis sensor/effector fusion protein WspE	0.301 (3.33E-04)	–	Down/–
HZ99_RS10665	WP_038442898.1	Chemotaxis protein WspD	0.321 (7.73E-03)	–	Down/–
HZ99_RS10670	WP_038442899.1	Chemotaxis protein WspC	0.306 (2.22E-02)	–	Down/–
HZ99_RS10675	WP_038442901.1	Chemotaxis protein WspB	0.292 (2.76E-04)	–	Down/–
HZ99_RS10680	WP_038442902.1	Chemotaxis transducer WspA	0.251 (1.05E-04)	0.812 (6.07E-03)	Down/Down
HZ99_RS14415	WP_017739508.1	PrkA family serine protein kinase	0.014 (3.29E-16)	0.097 (2.78E-04)	Down/Down
HZ99_RS14470	WP_038443829.1	Bifunctional diguanylate cyclase/phosphodiesterase	0.044 (2.50E-09)	–	Down/–
HZ99_RS15265	WP_038444050.1	ArsR family transcriptional regulator	–	0.578 (2.84E-03)	–/Down
HZ99_RS16305	WP_038444414.1	DNA-binding response regulator	0.512 (1.97E-02)	0.795 (9.15E-03)	Down/Down
HZ99_RS19375	WP_038445316.1	Two-component system response regulator NtrC	0.354 (2.64E-04)	0.759 (1.09E-02)	Down/Down
HZ99_RS20755	WP_038445694.1	PAS domain-containing sensor histidine kinase	0.037 (1.74E-09)	–	Down/–
HZ99_RS20770	WP_038445699.1	DNA-binding response regulator CitB	0.129 (2.11E-07)	0.574 (2.15E-03)	Down/Down
HZ99_RS23975	WP_038446534.1	Bifunctional diguanylate cyclase/phosphodiesterase	0.073 (9.68E-06)	–	Down/–
HZ99_RS24460	WP_038446651.1	RNA polymerase sigma-H factor AlgU	–	1.458 (1.78E-02)	-/Up
HZ99_RS25335	WP_038446976.1	Anti-anti-sigma factor HsbA	0.087 (1.88E-04)	0.182 (4.82E-05)	Down/Down
HZ99_RS25340	WP_038446978.1	HptB-dependent secretion and biofilm regulator HsbR	0.090 (2.23E-03)	–	–
HZ99_RS25765	WP_038447108.1	Methyl-accepting chemotaxis protein	–	0.565 (2.85E-03)	–/Down
**POLYSACCHARIDE METABOLISM**					
HZ99_RS00070	WP_038440366.1	Polysaccharide biosynthesis protein PslL	0.215 (2.35E-08)	–	Down/–
HZ99_RS00075	WP_038440367.1	Acyltransferase	0.169 (2.82E-11)	–	Down/–
HZ99_RS00080	WP_038440369.1	Polysaccharide biosynthesis protein PslJ	0.161 (1.58E-11)	–	Down/–
HZ99_RS00085	WP_038440370.1	Polysaccharide biosynthesis protein PslI	0.127 (4.49E-14)	0.619 (1.95E-02)	Down/Down
HZ99_RS00090	WP_038440371.1	Polysaccharide biosynthesis protein PslH	0.136 (1.51E-13)	–	Down/–
HZ99_RS00095	WP_038440372.1	Polysaccharide biosynthesis protein pslG	0.098 (6.06E-18)	0.577 (1.43E-02)	Down/Down
HZ99_RS00100	WP_038440373.1	Polysaccharide biosynthesis protein PslF	0.117 (2.05E-13)	0.684 (1.25E-02)	Down/Down
HZ99_RS00105	WP_038440374.1	Polysaccharide biosynthesis protein PslE	0.102 (1.81E-16)	0.590 (4.52E-03)	Down/Down
HZ99_RS00110	WP_038440375.1	Polysaccharide biosynthesis protein PslD	0.085 (6.34E-17)	–	Down/–
HZ99_RS00115	WP_038440377.1	Polysaccharide biosynthesis protein PslC	0.092 (2.69E-16)	0.526 (1.20E-03)	Down/Down
HZ99_RS00120	WP_038440378.1	Polysaccharide biosynthesis protein PslB	0.107 (9.57E-14)	0.593 (2.05E-02)	Down/Down
HZ99_RS00125	WP_038440379.1	Polysaccharide biosynthesis protein PslA	0.078 (3.34E-16)	–	Down/–
HZ99_RS03630	WP_038441389.1	Glycogen synthase GlgA	0.355 (1.23E-04)	–	Down/–
HZ99_RS03635	WP_038441390.1	Malto-oligosyltrehalose trehalohydrolase TreZ	0.266 (7.44E-06)	–	Down/–
HZ99_RS03640	WP_038441391.1	4-alpha-glucanotransferase MalQ	0.405 (2.76E-03)	–	Down/–
HZ99_RS03645	WP_038441393.1	Malto-oligosyltrehalose synthase TreY	0.234 (1.78E-07)	–	Down/–
HZ99_RS03650	WP_038441395.1	Hypothetical protein	0.365 (1.87E-03)	–	Down/–
HZ99_RS03655	WP_038441396.1	Glycogen debranching enzyme GlgX	0.299 (2.46E-05)	0.747 (4.89E-02)	Down/Down
HZ99_RS03670	WP_038441402.1	1,4-alpha-glucan branching protein GlgB	0.210 (1.57E-07)	–	Down/–
HZ99_RS03675	WP_038441404.1	Maltose alpha-D-glucosyltransferase TreS	0.298 (1.60E-05)	–	Down/–
HZ99_RS03680	WP_038441406.1	Alpha-1,4-glucan—maltose-1-phosphate maltosyltransferase GlgE	0.247 (6.95E-07)	–
HZ99_RS10715	WP_038442912.1	Phosphoethanolamine transferase OpgE	0.237 (2.95E-02)	0.553 (1.08E-02)	Down/Down
HZ99_RS19405	WP_038445324.1	Maltodextrin phosphorylase GlgP	0.190 (1.31E-09)	0.553 (5.91E-03)	Down/Down
HZ99_RS23185	WP_038446334.1	Trehalose permease IIC protein		0.554(5.91E-03)	–/Down
**INTRACELLULAR SECRETION AND EXTRACELLULAR STRUCTURES**					
HZ99_RS00355	WP_080727762.1	Type V secretory pathway, adhesin AidA	0.032 (3.29E-03)	-	Down/–
HZ99_RS03975	WP_008603564.1	FapF	0.017 (3.21E-08)	0.468 (2.66E-04)	Down/Down
HZ99_RS20775	WP_038445702.1	Hypothetical protein	0.029 (6.35E-15)	–	Down/–
HZ99_RS20780	WP_038445704.1	Type II secretion system protein TadD	0.018 (8.12E-10)	–	Down/–
HZ99_RS20785	WP_038445705.1	Pilus assembly protein TadC	0.021 (2.26E-09)	–	Down/–
HZ99_RS20790	WP_038445707.1	Flp pilus assembly protein TadB	0.022 (5.07E-09)	–	Down/–
HZ99_RS20795	WP_038445708.1	ATPase TadA	0.020 (4.85E-11)	0.100 (5.84E-04)	Down/Down
HZ99_RS20800	WP_038445709.1	Flp pilus assembly protein TadZ	0.016 (3.03E-11)	0.216 (2.83E-03)	Down/Down
HZ99_RS20805	WP_038445710.1	Type II/III secretion system protein RcpA	0.017 (2.35E-08)	–	Down/–
HZ99_RS20810	WP_038445713.1	Flp pilus assembly protein RcpC	0.010 (1.38E-14)	0.217 (1.61E-03)	Down/Down
HZ99_RS20815	WP_038445716.1	Flp family type IVb pilin	0.040 (9.64E-13)	–	Down/–
HZ99_RS20820	WP_038445718.1	Response regulator	0.043 (7.89E-12)	–	Down/–
HZ99_RS20825	WP_038445719.1	Flp family type IVb pilin	0.059 (6.93E-15)	–	Down/–
HZ99_RS20830	WP_038445722.1	Hemolysin activation/secretion protein FhaC	0.017 (6.11E-10)	0.380 (1.97E-03)	Down/Down
HZ99_RS21155	WP_024015038.1	Type II secretory pathway, component ExeA (predicted ATPase)	0.088 (2.31E-11)	0.201 (3.09E-04)	Down/Down
HZ99_RS24960	WP_038446836.1	HlyD family type I secretion periplasmic adaptor subunit	0.073 (2.27E-09)	–	Down/–
HZ99_RS24965	WP_038448190.1	Type I secretion system permease/ATPase	0.085 (7.92E-11)	–	Down/–
HZ99_RS24970	WP_038446837.1	Type I secretion outer membrane protein, TolC family	0.019 (1.61E-04)	0.310 (2.86E-04)	Down/Down
**CELL WALL BIOGENESIS**					
HZ99_RS02640	WP_038441173.1	LPS O-antigen chain length determinant protein	0.297 (3.08E-06)	0.800 (3.04E-02)	Down/Down
HZ99_RS09290	WP_038442547.1	UDP 4-amino-4-deoxy-L-arabinose aminotransferase	0.064 (8.84E-04)	–	Down/–
HZ99_RS09295	WP_038442548.1	UDP 4-deoxy-4-formamido-L-arabinose transferase ArnC	0.040 (2.47E-04)	–	Down/–
HZ99_RS09300	WP_038442549.1	Bifunctional UDP-glucuronic acid oxidase/UDP-4-amino-4-deoxy-L-arabinose formyltransferase ArnA	0.087 (2.13E-03)	0.202 (1.00E-03)	Down/Down
HZ99_RS09305	WP_038442550.1	4-deoxy-4-formamido-L-arabinose-phosphoundecaprenol deformylase ArnD	0.069 (1.15E-06)	–	Down/–
HZ99_RS09310	WP_038442551.1	4-amino-4-deoxy-L-arabinose transferase ArnT	0.069 (5.68E-04)	–	Down/–
HZ99_RS09315	WP_038442552.1	4-amino-4-deoxy-L-arabinose-phospho-UDP flippase ArnE	0.175 (4.61E-02)	–	Down/–
HZ99_RS09325	WP_038442554.1	UDP-glucose 6-dehydrogenase	0.100 (4.32E-06)	0.442 (3.52E-03)	Down/Down
HZ99_RS26590	WP_038447400.1	Glycosyl transferase	–	0.443 (1.87E-02)	–/Down
**STRESS RESPONSES**					
HZ99_RS02240	WP_038441046.1	Peroxiredoxin AhpC	–	0.809 (2.96E-02)	–/Down
HZ99_RS13955	WP_038443738.1	Catalase KatE	0.194 (5.59E-05)	0.469 (2.68E-03)	Down/Down
HZ99_RS16790	WP_028618516.1	GlsB/YeaQ/YmgE family stress response membrane protein	0.165 (2.28E-04)	–	Down/–
HZ99_RS17150	WP_038444652.1	RND divalent metal cation efflux transporter CzcA	0.379 (2.52E-04)	–	Down/–
HZ99_RS17155	WP_019334917.1	RND divalent metal cation efflux membrane fusion protein CzcB precursor	0.293 (3.53E-02)	–	Down/–
HZ99_RS17205	WP_038444665.1	Copper resistance protein A precursor PcoA	0.185 (9.81E-03)	0.621 (1.29E-03)	Down/Down
HZ99_RS17210	WP_038444668.1	Copper resistance protein B precursor PcoB	0.195 (3.68E-02)	0.757 (2.90E-02)	Down/Down
HZ99_RS17635	WP_038444793.1	Peroxiredoxin OsmC	0.116 (1.73E-06)	0.251 (4.43E-03)	Down/Down
HZ99_RS18120	WP_038444941.1	Multidrug export protein EmrA	0.156 (4.07E-11)	–	Down/–
HZ99_RS18555	WP_038445116.1	RND triclosan efflux membrane fusion protein, TriA	0.210 (1.11E-02)	–	Down/–
HZ99_RS18560	WP_038445117.1	RND triclosan efflux membrane fusion protein, TriB	0.235 (1.13E-03)	0.550 (1.04E-02)	Down/Down
HZ99_RS18565	WP_038445119.1	RND triclosan efflux membrane fusion protein, TriC	0.184 (4.00E-04)	–	Down/–
HZ99_RS22450	WP_038446132.1	Glutathione peroxidase	–	0.763 (0.00123)	–/Down
HZ99_RS26805	WP_029298005.1	Universal stress protein UspA	0.248 (1.72E-07)	–	Down/–
**AMINO ACID AND BIOGENIC AMINE METABOLISM**					
HZ99_RS02405	WP_038441100.1	Spermidine synthase	0.115 (1.56E-05)	–	Down/–
HZ99_RS04195	WP_038441535.1	Lysine decarboxylase	0.191 (1.23E-05)	–	Down/–
HZ99_RS06735	WP_038441955.1	Methionine gamma-lyase	–	0.482 (8.69E-03)	–/Down
HZ99_RS07550	WP_038442095.1	Agmatine deiminase	0.125 (6.06E-07)	–	Down/–
HZ99_RS10175	WP_038442810.1	Arginine deiminase	–	0.797 (7.34E-03)	–/Down
HZ99_RS10185	WP_038442812.1	Carbamate kinase ArcC	0.468 (4.14E-02)	0.803 (2.35E-03)	Down/Down
HZ99_RS14340	WP_038443794.1	Spermidine/putrescine ABC transporter substrate-binding protein	–	0.524 (2.47E-02)	–/Down
HZ99_RS14900	WP_038443918.1	Glycine cleavage system protein R	0.194 (9.81E-05)	0.391 (9.62E-03)	Down/Down
HZ99_RS15270	WP_038444052.1	Methionine adenosyltransferase	0.482 (1.05E-02)	0.580 (1.57E-03)	Down/Down
HZ99_RS20705	WP_038445679.1	Arginine decarboxylase SpeA	0.397 (4.55E-04)	0.821 (1.61E-02)	Down/Down
HZ99_RS22590	WP_038446180.1	Glu/Leu/Phe/Val dehydrogenase	0.083 (2.60E-10)	0.293 (3.62E-04)	Down/Down
HZ99_RS26135	WP_038447234.1	Spermidine/putrescine ABC transporter ATP-binding protein	0.163 (3.69E-04)	–	Down/–
HZ99_RS26140	WP_029292823.1	ABC-type spermidine/putrescine transport system, permease component II	0.169 (1.39E-02)	–	Down/–
HZ99_RS26155	WP_038447240.1	Spermidine/putrescine-binding periplasmic protein	0.110 (1.70E-02)	0.461 (8.83E-03)	Down/Down
**OXIDATIVE PHOSPHORYLATION**					
HZ99_RS17715	WP_038444813.1	Cytochrome b559 subunit alpha	0.029 (1.72E-07)	0.194 (9.29E-05)	Down/Down
HZ99_RS17720	WP_038444815.1	Cytochrome c oxidase subunit I	0.035 (1.81E-03)	–	Down/–
HZ99_RS17725	WP_038444816.1	Cytochrome c oxidase assembly protein	0.032 (9.73E-04)	–	Down/–
HZ99_RS17730	WP_038444818.1	Cytochrome c oxidase subunit III	0.044 (2.36E-04)	–	Down/–
HZ99_RS17740	WP_038444821.1	Cytochrome oxidase biogenesis protein Surf1,facilitates heme A insertion	0.026 (2.98E-07)	–	Down/–
HZ99_RS17745	WP_038448109.1	Hypothetical protein	0.042 (7.68E-06)	–	Down/–
HZ99_RS17750	WP_038444822.1	Cytochrome b561	0.051 (4.43E-05)	–	Down/–
HZ99_RS17755	WP_038444823.1	Protoheme IX farnesyltransferase	0.044 (4.04E-04)	–	Down/–
HZ99_RS23395	WP_038446389.1	NADH:flavin oxidoreductase/NADH oxidase	0.017 (4.36E-07)	0.275 (5.50E-06)	Down/Down
**SULFUR METABOLISM**					
HZ99_RS15945	WP_003213899.1	Transporter	6.011 (3.59E-04)		Up/–
HZ99_RS15950	WP_038444290.1	Aliphatic sulfonates ABC transporter ATP-binding protein SsuB	5.495 (5.07E-04)		Up/–
HZ99_RS15955	WP_038444293.1	Alkanesulfonate transporter permease subunit SsuC	4.988 (3.08E-02)		Up/–
HZ99_RS18395	WP_038445051.1	Sulfate-binding protein precursor	-	1.394 (4.14E-02)	–/Up
HZ99_RS18400	WP_032856532.1	Sulfate ABC transporter permease subunit CysT	2.407 (1.39E-02)		Up/–
HZ99_RS18410	WP_038445056.1	Sulfate ABC transporter ATP-binding protein CysA	2.391 (6.81E-03)		Up/–
HZ99_RS18930	WP_038445174.1	Taurine dioxygenase TauD	2.522 (2.42E-02)		Up/–
HZ99_RS18940	WP_038445178.1	Taurine transporter ATP-binding subunit TauB	–	1.597 (2.31E-04)	–/Up
HZ99_RS18945	WP_038445179.1	Taurine ABC transporter substrate-binding protein TauA	–	1.319 (2.97E-02)	–/Up
HZ99_RS27240	WP_038447698.1	Methanesulfonate sulfonatase MsuD	–	1.447 (6.49E-03)	–/Up

a *CDSs belonging to a putative operon are marked with a box*.

b, c, d *The symbol “–” indicates that the expression of the gene was not detected or was not significantly different between the rpoS mutant and wild-type*.

### Transcription Factors and Signal Transduction

According to the COG functional category, except for the CDSs categorized as COG S or COG R, Group K and Group T were the two top groups (Figure 2). The GO and KEGG analyses also showed that signal transduction and two-component system were significantly enriched (Figures 3, 4), and most of these CDSs were downregulated at the mRNA and protein levels in the *rpoS* mutant. As shown in Table 1, the operon RS01140-35 encoding homologs of the quorum-sensing system RhlR-RhlI was significantly downregulated in the *rpoS* mutant. This agrees with our previous result that RpoS positively regulates AHL synthesis (Liu et al., 2018). The adjacent operon RS01150-45 (*rhlAB*) that is involved in rhamnolipid biosurfactant synthesis, was also positively regulated by RpoS. In *P*. *aeruginosa*, RhlR-RhlI regulates the *rhlAB* operon and affects rhamnolipid biosurfactant synthesis (Reis et al., 2011). Biosurfactants can play an important role in the bacterial spoilage process. *P*. *fluorescens* leads to the spoilage of aerobically stored chicken meat by producing biosurfactants that can make nutrients more freely available and providing the strains producing them with a competitive advantage (Mellor et al., 2011). The operon RS10680-50 (*wspABCDEFR*), coding for a chemosensory system, and the regulation proteins RsmA, HsbA, and HsbR have all been observed to regulate biofilm formation of *P*. *aeruginosa* (Hickman et al., 2005; Hsu et al., 2008; Irie et al., 2010). RpoS also positively regulates the response regulator NtrC of the two-component system NtrB-NtrC, which is involved in nitrogen metabolism. In *P*. *fluorescens* SBW25, deletion of *ntrC* eliminates the degradation and the utilization of 28 nitrogen substrates (Zhang and Rainey, 2008). Several regulators related to stress resistance are regulated by RpoS, such as BasR and RpoE (Haines-Menges et al., 2014; Rubin et al., 2015). In addition, many CDSs coding for protein kinases and diguanylate cyclase/phosphodiesterase were downregulated in the *rpoS* mutant, and these enzymes may play an important role in signal transduction. Accordingly, RpoS is a global regulator showing direct or indirect control of many secondary transcription factors and signal transducing proteins.

### Polysaccharide Metabolism

According to the COG analysis and GO enrichment analysis, a large part of DECs and DEPs were involved in the synthesis of various polysaccharides. There was a large operon, RS00125-070, that showed strong downregulation in the *rpoS* mutant at the mRNA and protein levels (Table 1). This operon is homologous to the *psl* operon of *P*. *aeruginosa*, a locus encoding a potential exopolysaccharide that is essential for biofilm formation (Jackson et al., 2004). The operon contained 12 co-transcribed CDSs. Among the 12 CDSs, 8 CDSs were enriched in the biofilm pathway of *P*. *aeruginosa* (Figure 4; Supplementary Table S10). As shown in Figure 6, *P*. *fluorescens* UK4 formed red and wrinkled macrocolony biofilms on agar plates containing Congo red, whereas the *rpoS* mutant did not. Congo red binds extracellular matrix components of bacteria, such as polysaccharides and amyloid adhesins (Larsen et al., 2007; Irie et al., 2010). Moreover, the results of TEM showed that most cells of UK4 macrocolony biofilms were surrounded by extracellular matrix, which was more disordered and much less than in the *rpoS* mutant. Similar to the present results, Irie et al. (2010) found that RpoS is a positive transcriptional regulator of *psl* gene expression in *P*. *aeruginosa* PAO1, and Psl overproduction confers rugose and red small colony variant morphology on Congo red plates. In *E*. *coli*, RpoS regulates the formation of wrinkled and red macrocolony biofilms on Congo red plates by controlling the production of cellulose and curli fibers (Mika and Hengge, 2014). Our results suggest that RpoS possibly regulates the formation of macrocolony biofilm by positively regulating the expression of *psl* genes coding for polysaccharide synthesis in *P*. *fluorescens*. Bacteria in biofilms are generally well-protected against environmental stress, and consequently, they are extremely difficult to detect and eradicate in food processing environments.

In addition, several CDSs and proteins were significantly enriched in the starch and sucrose metabolism pathway (Figures 4, 5). They are associated with glycogen and trehalose metabolism, and are mainly located in two tandem operons, RS03630-55 and RS03680-70. These DECs were downregulated in the *rpoS* mutant, suggesting that RpoS positively regulated glycogen and trehalose metabolism. These results were similar to a previous report showing that RpoS regulates the expression of the carbon storage genes for the biosynthesis and degradation of glycogen and the osmoprotectant trehalose in *Salmonella* (Lévi-Meyrueis et al., 2014). Glycogen and trehalose are two important storage metabolites, and their amounts change in response to a number of environmental stresses, such as osmotic, oxidative, and cold stress (Dalmasso et al., 2012; Kobayashi et al., 2015). Additionally, the glycogen biosynthesis enzymes GlgC, GlgA, and GlgP have been observed to play key roles in the formation of biofilms in *E*. *coli*. Stress resistance and biofilm formation are important factors for the spoilage activities of bacteria (Van Houdt and Michiels, 2010). Thus, RpoS might regulate spoilage activities by controlling polysaccharide synthesis in *P*. *fluorescens*.

### Intracellular Secretion and Extracellular Structures

Two tandem operons, RS20810-20775 and RS20815-20825, related to pilus formation, were notably downregulated in the *rpoS* mutant at the mRNA and protein levels (Table 1). The operon RS20810-775 contains 8 CDSs, including 2 CDSs coding for proteins RcpC and RcpA, 5 CDSs coding for TadZ, TadA, TadB, TadC, and TadD, and 1 coding for a hypothetical protein. The other operon RS20815-25 contains 3 CDSs, among which RS20815 and RS20825 code for Flp family type IVb pilins, and RS20820 codes for a response regulator. The homologs of the two operons are both required for the formation of adhesive Flp pili in *Aggregatibacter actinomycetemcomitans* (Clock et al., 2008). In addition, RS03975 codes for a β-barrel membrane pore, which is analogous to the *Pseudomonas* FapF protein involved in the transport of amyloid-like fimbriae monomers (Rouse et al., 2018). The *fapF* gene belongs to the operon *fapABCDEF* related to amyloid-like fimbriae formation (Zeng et al., 2015). However, according to our results, only the *fapF* gene of the operon was regulated by RpoS, suggesting that *fapF* may have an independent promoter. Flp pili and amyloid-like fimbriae have been reported to be essential for adherence, colonization, and biofilm formation in many bacteria (de Bentzmann et al., 2006; Romero et al., 2010; Stenvang et al., 2016), and they may also contribute to macrocolony biofilm formation in addition to exopolysaccharide in *P*. *fluorescens*. Pilus formation is likely another factor responsible for the spoilage activity of *P. fluorescens*.

### Cell Wall Biogenesis

Several genes associated with cell wall biogenesis were differentially expressed in the analyzed strains (Figure 2). In the functional group (COG M), RS02640, RS26590 and the operon RS09290-325 were remarkably downregulated in the *rpoS* mutant at the mRNA and protein levels. RS02640 codes for an LPS O-antigen chain length determinant protein, and RS26590 codes for a glycosyl transferase involved in cell wall biosynthesis. It is noteworthy that in operon RS09290-315 there were 5 CDSs enriched in the cationic antimicrobial peptide (CAMP) resistance pathway (Figure 5; Supplementary Table S10). The five CDSs code for homologs of ArnA, ArnC, ArnD, ArnE, and ArnT in *E*. *coli*, which contribute to the biosynthesis of UDP-L-Ara4FN and transfer of the L-Ara4N moiety to lipid A (Breazeale et al., 2005). In *E*. *coli* and *Salmonella typhimurium*, the addition of L-Ara4N groups to lipid A under the control of the transcription factor BasR (PmrA) is required for maintaining resistance to certain cationic antimicrobial peptides (Yan et al., 2007; Rubin et al., 2015). This agrees with our result showing that the expression of the *basR* (*pmrA*) gene was also downregulated in the *rpoS* mutant (Table 1). The expression of CDSs with functions related to cell wall synthesis and maintenance is involved in resistance to external stresses that damage the cell envelope (Liu et al., 2017). Our results indicate that RpoS might contribute to the formation of resistance to cell wall stress conditions.

### Stress Responses

As expected, many of the identified downregulated CDSs and proteins were those that are important for stress response. For example, the *rpoS* mutation resulted in decreased expression of CDSs coding for antioxidant enzymes (AhpC, KatE, OsmC), RND transporters (TriABC, CzcAB), and other stress related proteins (PcoAB, UspA) (Table 1). RpoS controls the antioxidant enzymes that are also observed in *E*. *coli, Salmonella*, and *B. pseudomallei* (Dong and Schellhorn, 2009; Osiriphun et al., 2009; Lévi-Meyrueis et al., 2014). Levels of reactive oxygen species can increase when bacteria are exposed to certain types of environmental stresses, such as heat, ethanol, ultraviolet radiation, or antibiotics, and the oxidative injury to cells can be reduced by increased antioxidant enzyme activities (Liu et al., 2012; El-Halfawy and Valvano, 2014). The Resistance-Nodulation-Cell Division (RND) transporters mainly contribute to resistance to antimicrobial agents (Venter et al., 2015). The RND pump expression is regulated in response to external stress factors such as reactive oxygen species, membrane damaging agents or ribosome blocking substances (Dreier and Ruggerone, 2015). The copper resistance proteins PcoA and PcoB contribute to copper resistance in *E. coli* and *Pseudomonas* (Chihomvu et al., 2015). The universal stress protein UspA is involved in oxidative stress defense, and the induction of the UspA protein is independent of RpoS in *E*. *coli* (Kim et al., 2012). However, our results indicated that the expression of UspA was positively regulated by RpoS at the mRNA level in *P. fluorescens*. Our previous work indicated that RpoS affects the resistance of *P. fluorescens* to several stress conditions (Liu et al., 2018). Taken together, RpoS may regulate the resistance of *P. fluorescens* to the stress conditions by controlling the expression of these stress-related CDSs and proteins.

### Amino Acid and Biogenic Amine Metabolism

Microbial growth and metabolism are a major cause of food spoilage that produces ammonia and biogenic amines such as putrescine, histamine, and cadaverine with unpleasant and unacceptable off-flavors (Ghaly et al., 2010). In the *rpoS* mutant of *P*. *fluorescens*, the expression of a large group of CDSs related to amino acid transport and metabolism (COG E) was significantly downregulated at the mRNA and protein levels (Figure 2). Many of these CDSs are involved in producing ammonia and biogenic amines, and typical DECs and DEPs are shown in Table 1. RS06735, RS07550, RS10175, RS14900, and RS22590 encode proteins related to the generation of ammonia, while RS02405, RS04195, RS07550, and RS20705 code for enzymes resulting in the production of spermidine, cadaverine, N-carbamoylputrescine, and agmatine, respectively. In addition, RS14340, RS26135, RS26140, and RS26155 code for proteins involved in the transport of spermidine or putrescine. Similarly, RpoS activates genes for transport and degradation of amino acids in *P*. *aeruginosa, Salmonella*, and *E*. *coli* (Schuster et al., 2004; Dong and Schellhorn, 2009; Lévi-Meyrueis et al., 2014). Our previous study showed that the *rpoS* mutant of *P*. *fluorescens* significantly reduces TVB-N production in sterilized salmon juice (Liu et al., 2018), which might be caused by downregulating the expression of CDSs related to the production of ammonia and biogenic amines in the mutant. So RpoS may play an important role in the spoilage potential of *P*. *fluorescens* by controlling the expression of CDSs related to amino acid and biogenic amine metabolism.

### Oxidative Phosphorylation

The KEGG analyses showed that 10 downregulated CDSs and 1 upregulated CDS were enriched in the oxidative phosphorylation pathway (Figure 4; Supplementary Table S10). It is worthy to note that two tandem operons, RS17715-30 and RS17740-55, that code for proteins contributing to the respiratory chain were notably downregulated in the *rpoS* mutant at the mRNA and protein levels. Consistent with this, RpoS also positively regulates CDSs coding for cytochrome c oxidase in *P*. *aeruginosa* and *Geobacter sulfurreducens* (Schuster et al., 2004; Núñez et al., 2006). Lindqvist et al. (2000) indicated that the respiratory oxidases can protect *E*. *coli* K12 from oxidative stress. However, negative effects of RpoS on the respiratory chain have been observed in *Salmonella* (Lévi-Meyrueis et al., 2014). The effects of RpoS on oxidative phosphorylation are species-dependent.

### Sulfur Metabolism

As mentioned above, RpoS mainly functions as a positive regulator. Only a small portion of CDSs were negatively regulated by RpoS, and most of these negatively regulated CDSs showed a smaller change in expression levels compared to the positively regulated CDSs at both the mRNA and protein levels (Supplementary Tables S6, S7). However, according to the KEGG enrichment analyses, the CDSs enriched in sulfur metabolism were all negatively regulated by RpoS and are all related to the utilization of sulfonate and sulfate. As a sigma factor, negative control showed by RpoS is likely to be an indirect regulation, probably resulting from sigma factor competition (Farewell et al., 1998).

## Conclusions

In this study, the RpoS regulon was identified by a combined transcriptome and proteome analysis of *P*. *fluorescens* wild-type strain UK4 and its *rpoS* mutant in the stationary phase. This analysis showed that RpoS regulated the expression of a large set of CDSs at the mRNA and protein levels, mainly including those related to polysaccharide metabolism, intracellular secretion and extracellular structures, cell wall biogenesis, stress responses, ammonia, and biogenic amine metabolism. These cell processes may contribute to biofilm formation, stress resistance and spoilage activities of *P*. *fluorescens*, and may be also regulated by the transcription factors and signal transduction dependent on RpoS. In addition, several other pathways, such as oxidative phosphorylation, sulfur metabolism and so on, were also affected by RpoS, showing the roles of RpoS in cell processes are diverse. The present findings expand our knowledge about the regulatory mechanisms of bacterial spoilage. RpoS and the pathways regulated by RpoS may be used as potential targets for new food preservative screening, or as molecular markers in assessment of microbial food safety and food quality.

## Author Contributions

XL conceived and designed the experiments, carried out the experiments, analyzed the data and wrote the manuscript. JX, JZ, and PD participated in performing experiments, and analyzing and interpreting the data. AS participated in designing the experiments, analyzing the data, and revising the manuscript. XL and AS supervised the project as co-correspondence. All the authors have read and approved the final version of the manuscript.

### Conflict of Interest Statement

The authors declare that the research was conducted in the absence of any commercial or financial relationships that could be construed as a potential conflict of interest.

## Abbreviations

AHL: Acylated homoserine lactone
COG: Cluster of orthologous groups
DECs: Differentially expressed coding sequences
DEPs: Differently expressed proteins
DTT: Dithiothreitol
EDTA: Ethylene diamine tetraacetic acid
FPKM: Fragments per kilobase of transcript sequence per millions base pairs sequenced
GO: Gene ontology
HPLC: high-performance liquid chromatography
KEGG: Kyoto encyclopedia of genes and genomes
MS: Mass spectrometry
NB: Nutrient broth
qRT-PCR: quantitative real-time PCR
RNA-seq: RNA sequencing
TEAB: Tetraethyl-ammonium bromide
TEM: Transmission electron microscopy
TMT: Tandem mass tag
UPLC: Ultra performance liquid chromatography.
