# Supplementary material for: Combined Transcriptome and Proteome Analysis of RpoS Regulon Reveals Its Role in Spoilage Potential of Pseudomonas fluorescens
Source: Front Microbiol. 2019 Feb 6;10:94. doi: 10.3389/fmicb.2019.00094 (PMC6372562; doi:10.3389/fmicb.2019.00094)
Supplement: Supplementary file 2 [file Table_2.docx]

**Supplementary Table S2** List of primers used for qRT-PCR.

| CDS | Gene product description | Primer Sequences (5'to3') | Size (bp) |
| --- | --- | --- | --- |
| RS00070 | polysaccharide biosynthesis protein PslL | F: CCGATCAGCCGTACCCACAA | 140 |
|  |  | R: GCCCTGCCCCTCTATCAACAA |  |
| RS00125 | polysaccharide biosynthesis protein PslA | F: CCGACCATTTGGCGATGTATT | 155 |
|  |  | R: CGAGGAAGCCGTCAACGAGTA |  |
| RS00290 | DNA-binding response regulator | F: CGTGCAACCGTCAAGTACCTGT | 97 |
|  |  | R: GCTCCTCCCTGGCAATCTGTAA |  |
| RS00355 | Type V secretory pathway, adhesin AidA | F: CTGGCAACCTGACCCTGGAT | 89 |
|  |  | R: GCCACCGTAGCGGAACAACT |  |
| RS01135 | acyl-homoserine-lactone synthase RhlI | F: GGATTGGCTGGTCGCACAGA | 103 |
|  |  | R: CTGGACGCACAAGGTCAGGTTT |  |
| RS01150 | MFS transporter | F: GCCGCTGACGTATTCGAGCAT | 136 |
|  |  | R: CCATCTGGTTTCCGCCTCCT |  |
| RS01735 | LuxR family transcriptional regulator | F: CGGACCTGGTGGTACTGGACAT | 132 |
|  |  | R: GCTTGAGGTACAACGAAGGCTCA |  |
| RS02405 | spermidine synthase | F: CACCAACAGCATCTCGTGGAACT | 105 |
|  |  | R: CCGACGCAGAACATCGAAGACT |  |
| RS02950 | membrane protein | F: CGACCCAGCGCATTTACCAA | 117 |
|  |  | R: CGACCTTCAACACACCACCATT |  |
| RS03635 | malto-oligosyltrehalose trehalohydrolase TreZ | F: GCGACCACCTTATTGCTGATGT | 102 |
|  |  | R: GCCGTGAAGGTCGGTAAAGAA |  |
| RS03975 | FapF | F: GCTGTTTGGCTCGGTTTCCT | 91 |
|  |  | R: GACCTTGCCACCGACCTTCT |  |
| RS04195 | lysine decarboxylase | F: GGAGTGCCAGACGATCTTGTTG | 114 |
|  |  | R: GAACTGGGCTCGCTGTTGGAT |  |
| RS07550 | agmatine deiminase | F: CGCACCCAGTTGACCCTTGT | 102 |
|  |  | R: GGCACCCGCAATATCACCTTT |  |
| RS09290 | UDP 4-amino-4-deoxy-L-arabinose aminotransferase | F: GTCAAGCGTTCCTTCCGTTCTC | 86 |
|  |  | R: GATCCAGCCAGAGCGCAATACT |  |
| RS09820 | RNA polymerase subunit sigma-24, RpoE | F: GGCGACAGCACGGTTGAGTT | 92 |
|  |  | R: CCCAGACCGACTGGTCACAGA |  |
| RS09895 | methyl-accepting chemotaxis protein | F: CACGGTGGATTCGTTCGGAA | 89 |
|  |  | R: GGCCAGCTACATGCCAGTGTT |  |
| RS13905 | chloroperoxidase | F: CACGCTCATCATCCACGGTAG | 137 |
|  |  | R: GCTTGTGCGTGTCCGTCAAC |  |
| RS13955 | catalase KatE | F: GGGAATTTCAACGGATCACGAA | 102 |
|  |  | R: CCCGCGTGGTTTTGCACTTA |  |
| RS16320 | Glutathione S-transferase | F: GTCCCTGTCCGCTGCTACCTT | 117 |
|  |  | R: GCCGTAGGCGAGAAGCTGTTT |  |
| RS17635 | peroxiredoxin OsmC | F: CGCAGTTCGATGAGTTGAGCAA | 75 |
|  |  | R: GAGTGATGGTGGCGTTCAAGAC |  |
| RS17715 | cytochrome b559 subunit alpha | F: CGACCAAGACCCTGATCAACAT | 105 |
|  |  | R: CGTCCTGGCCCAGGTACTTGT |  |
| RS19375 | two-component response regulator NtrC | F: GCATGGGCCACCAGCTCTTT | 74 |
|  |  | R: CGCCTGAGCCACTCCAACAT |  |
| RS20705 | arginine decarboxylase SpeA | F: CGCCTGCTGCACTTTCACATG | 85 |
|  |  | R: CGTAGTAACGGATGGCTTCCTTGA |  |
| RS20810 | Flp pilus assembly protein RcpC | F: GGCACGATGATCTGCGATGT | 81 |
|  |  | R: CCCTGGGGATTATGTGGATGTA |  |
| RS22590 | Glu/Leu/Phe/Val dehydrogenase | F: CCGAACTGCTGGTGAGCGATA | 97 |
|  |  | R: GTGCTGAGCAAGGCGTCGTT |  |
| RS23395 | NADH:flavin oxidoreductase/NADH oxidase | F: GGGCACAGTGGGCATGATTAC | 108 |
|  |  | R: CAATACGGATCACGCAGCAGTT |  |
| RS24845 | aquaporin | F: CCACGACGCCACCAATGACT | 155 |
|  |  | R: CGGGCTTACCGTACTGACCAT |  |
| RS26135 | spermidine/putrescine ABC transporter ATP-binding protein | F: GGGGCTCATCGAGCAATAGCA | 153 |
|  |  | R: GCCATAAGCGGGTACAGGAAGT |  |
| RS26625 | competence protein ComEA | F: CCGTGACCTGTTTGGCATTG | 145 |
|  |  | R: GACTTGCCAATCCCCTTCACTT |  |
| RS26805 | universal stress protein UspA | F: GTTGTTCGCTGAGTCGGTGCT | 94 |
|  |  | R: GATGTTCGCCATTACGGTGTTC |  |
| 16S | internal control | F: GTCTCCTTAGAGTGCCCACCATTAC | 137 |
|  |  | R: GGTGCCTTCGGGAACATTGAGAC |  |
