# Supplementary material for: Combined Transcriptome and Proteome Analysis of RpoS Regulon Reveals Its Role in Spoilage Potential of Pseudomonas fluorescens
Source: Front Microbiol. 2019 Feb 6;10:94. doi: 10.3389/fmicb.2019.00094 (PMC6372562; doi:10.3389/fmicb.2019.00094)
Supplement: Supplementary file 11 [file Data_Sheet_1.PDF]

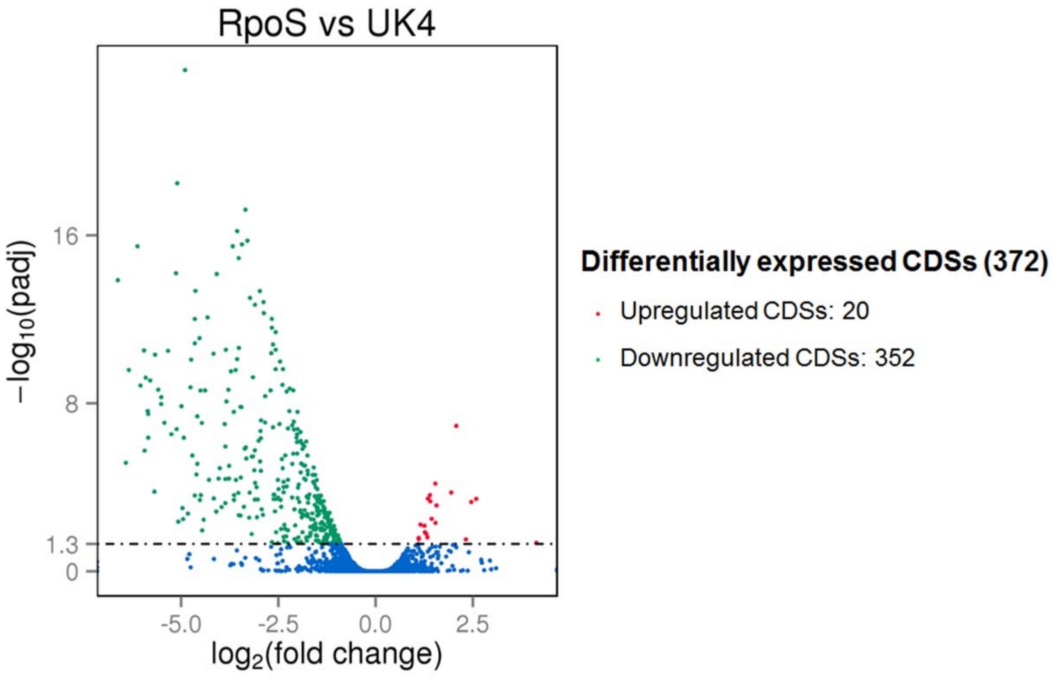

Supplementary Figure S1 DECs between the *rpoS* mutant and the wild-type strain. The horizontal axis represents fold changes of gene expression, and the vertical axis represents the statistically significance level. The red dots mean significantly upregulated genes and the green dots represent significantly downregulated genes.
