# Supplementary material for: Combined Transcriptome and Proteome Analysis of RpoS Regulon Reveals Its Role in Spoilage Potential of Pseudomonas fluorescens
Source: Front Microbiol. 2019 Feb 6;10:94. doi: 10.3389/fmicb.2019.00094 (PMC6372562; doi:10.3389/fmicb.2019.00094)
Supplement: Supplementary file 12 [file Data_Sheet_2.PDF]

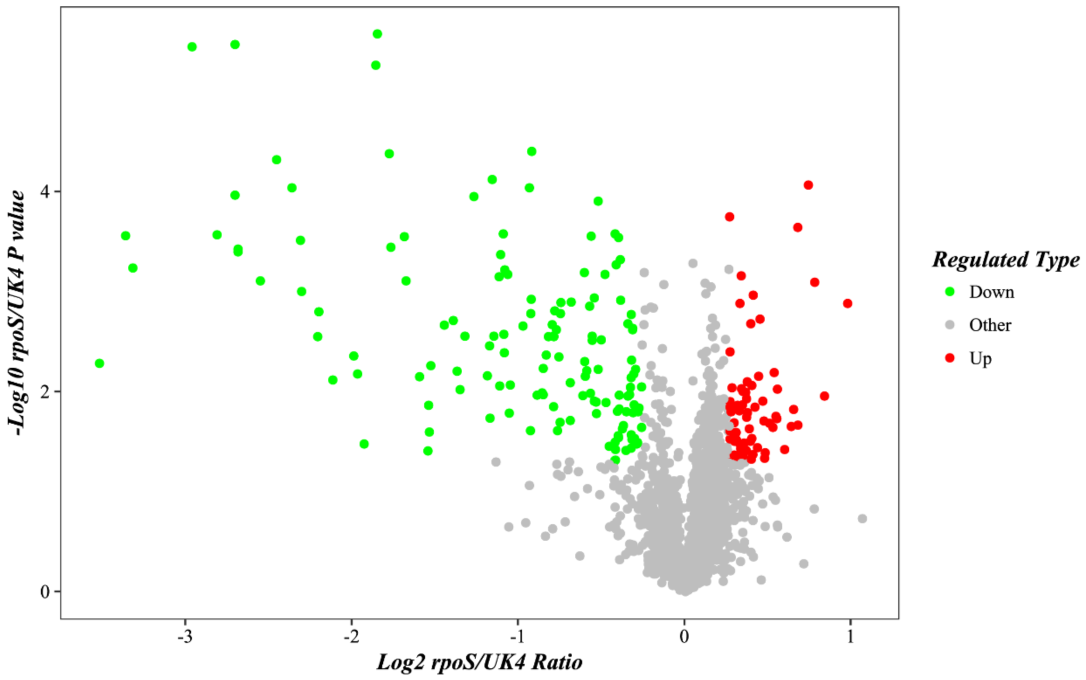

Supplementary Figure S2 DEPs between the *rpoS* mutant and the wild-type strain. The horizontal axis represents fold changes of protein expression, and the vertical axis represents the statistically significance level. The red dots mean significantly upregulated proteins and the green dots represent significantly downregulated proteins.
